# Erectile properties of the Rho-kinase inhibitor SAR407899 in diabetic animals and human isolated corpora cavernosa

**DOI:** 10.1186/1479-5876-10-59

**Published:** 2012-03-23

**Authors:** Fabio Guagnini, Mara Ferazzini, Marco Grasso, Salvatore Blanco, Tiziano Croci

**Affiliations:** 1 Research Center Sanofi-Midy, Exploratory Unit, sanofi R&D, Via G. Sbodio 2, Milano, Italy; 2 Department of Urology Ospedale San Gerardo, University Mi-Bicocca, Via Pergolesi 33, Monza, Italy

## Abstract

**Background:**

RhoA-Rho kinase complex contributes to keep the cavernosus smooth muscle contracted and its inhibition is considered a potential strategy for the therapy of erectile dysfunction (ED).

**Methods:**

We compared the effects of SAR407899, the Rho-kinase inhibitor Y-27632 and the PDE5 inhibitor sildenafil for their ability to relax corpus cavernosum strips contracted with phenylephrine in healthy and diabetic animals. Strips were obtained from WKY, spontaneous hypertensive (SHR), control CD, and diabetic CD rats, humans, control and diabetic rabbits. Diabetes was induced by streptozotocin or alloxan injection. *In vivo *penile erection (length) induced by drugs was measured in conscious rabbits.

**Results:**

SAR407899 dose-dependently relaxed the pre-contracted corpora cavernosa in all species, with similar potency and efficacy in healthy vs diabetic rats, WKY vs SHR rats, healthy vs diabetic rabbits (IC_50 _range from 0.05 to 0.29 μM, Emax range 89 to 97%). In the presence of the NO-synthase (NOS) inhibitor, L-NAME, the SAR407899 response did not decrease in any of the species or experimental conditions. The effect was confirmed in human strips where sildenafil was significantly less potent and effective, with IC_50 _respectively 0.13 and 0.51 μM; Emax 92 and 43%. Unlike SAR407899, the potency and efficacy of sildenafil and Y27632 were significantly reduced by diabetes and L-NAME. *In vivo*, SAR407899 dose-dependently induced rabbit penile erection, with greater potency and longer duration of action than sildenafil. Sildenafil, but not SAR407899, was less effective in alloxan-induced diabetes.

**Conclusion:**

The induction of penile erection by SAR407899, unlike that by sildenafil, is largely independent of e-NO activity. This suggests its use in erectile dysfunction for diabetic and hypertensive patients where e-NO activity is impaired.

## Background

Epidemiological studies indicate that about 50% of men aged between 40 and 70 years have some degree of erectile dysfunction (ED) which is often related to poor health or chronic illnesses such as atherosclerosis, diabetes mellitus, depression and hypogonadism [1,2]. ED may also be induced by drugs such as antidepressants, antihypertensives, viral protease inhibitors and antifungal agents or, less frequently, by physical trauma impairing either the penile arterial supply or spinal cord function [1,3,4].

Since the arrival of sildenafil in 1998, the type 5 phosphodiesterase (PDE_5_) inhibitors have been the mainstay for the treatment of virtually any type of ED. Sildenafil citrate (Viagra^®^) was in fact rapidly followed by the introduction, on the worldwide market, of two more PDE_5 _inhibitors: vardenafil (Levitra^®^) and tadalafil (Cialis^®^), and others are in advanced stages of development [5]. This class of drugs also largely contributed to understanding the mechanisms involved in penile erection, which results from a complex interplay of neurotransmitters, neuromodulators, endocrine and intracellular messengers, at both the central and peripheral levels [6,7].

Erection is basically a spinal reflex that can be initiated by recruitment of penile afferents but also by visual, olfactory and imaginary stimuli. Peripherally, penile erection is a hemodynamic event tuned by neurovascular mechanisms mediated by relaxation of the smooth muscle cells of the corpus cavernosum and its arterioles. This is accompanied by increased arterial blood flow into trabecular spaces which, together with a block of venous outflow, leads to organ tumescence. At molecular level, the nitric oxide (NO) transmitter, locally synthesized by NO-synthase (NOS), rapidly diffuses into the muscle cells and, activating the intracellular enzyme guanylate cyclase, induces the synthesis of cyclic-guanylyl-monophosphate (cGMP). This second messenger eventually leads to smooth muscle relaxation by triggering a cascade of phosphorylation reactions and Ca^++^-dependent processes [8]. The PDE_5 _inhibitors promote smooth muscle relaxation and penile erection by raising the intracellular cGMP concentration through selectively inhibiting its enzymatic degradation by PDE_5 _[9].

Although essential for penile erection, NO-dependent cGMP is not the only signaling process responsible for penile smooth muscle relaxation. Other mechanisms regulating smooth muscle tonus in the penis may be the targets of new drugs for the treatment of ED [10]. Among the systems helping keep the cavernous smooth muscles contracted, the one involving RhoA-Rho-kinase has been studied most [11]. In the smooth muscle cell RhoA-Rho-kinase is an important signal transduction pathway composed of the GTP-binding protein, RhoA and its effector phosphorylating enzyme, Rho-kinase, which is activated by vasoconstricting agents such as angiotensin, noradrenaline, endothelin, and platelet-derived growth factor (PDGF) [12]. When activated, this system keeps the myosin light chain (MLC) phosphorylated (MLC-P) by inhibiting MLC phosphatase. This facilitates the interaction between myosin and actin and, by sensitizing Ca^++^, eventually promotes smooth muscle cell contraction (for review see [13]).

The enzyme Rho-kinase was highly expressed in human and rabbit cavernosal smooth muscle [14,15] and it's *in vivo *inhibition by a specific antagonist induced cavernous smooth muscle relaxation and improved erectile function, particularly in the aging rat [11,16]. The RhoA/Rho-kinase system is up-regulated in the cavernosal tissue of aged and spontaneously hypertensive (SHR) rats and rats with experimentally-induced diabetes [17-19]. Up-regulation of this system might contribute to the ED associated with aging and the clinical conditions mentioned above.

This view is supported by the improvement of ED observed in animal models after inhibition of RhoA/Rho-kinase [20,21]. Therefore inhibition of this enzymatic pathway by selective antagonists may prove useful for the therapy of ED, particularly when associated with clinical conditions such as metabolic syndrome and type II diabetes, where current therapies with typical PDE_5 _antagonists appear to be less satisfactory [22].

One attractive feature of this mechanistic approach is that the antagonism of RhoA/Rho-kinase stimulates penile erection through a pathway independent of the NOS-cGMP pathway [11] and a positive interaction should be expected between RhoA/Rho-kinase antagonists and PDE5 inhibitors. Preclinical studies indicate that combined treatment with these two types of drugs is likely to achieve a better erectile response than either drug separately [19]. RhoA/Rho-kinase antagonists also have therapeutic potential in hypertension [23].

In the present study we examined the effects of SAR407899, a novel selective RhoA/Rho-kinase inhibitor, *in vitro *on the corpus cavernosum isolated from diabetic, SHR rats, diabetic rabbits, and humans. L-nitro-arginine-methyl-ester (L-NAME) was used to investigate the role of NO in the drug responses in different experimental conditions. We also assessed *in vivo *the ability of SAR407899 to induce penile erection in diabetic rabbits, compared with the PDE_5 _inhibitor, sildenafil and the RhoA/Rho-kinase inhibitor Y-27632, which is widely used in experimental pharmacology [24].

## Methods

### Animals

Sexually mature male CD, SHR and WKY (250-600 g) rats and New Zealand white rabbits (3.5-4.0 kg) were housed in a room with controlled temperature (22 ± 1°C), humidity (55 ± 10%) and 12-h light-dark cycle for at least ten days before being used. Food and water were available ad libitum.

In rats diabetes was induced in our laboratories by a single intravenous injection (lateral caudate vein) of streptozotocin (STZ) (50 mg/kg); in rabbits it was induced in the Charles River Laboratories by a single intravenous injection of alloxan monohydrate (120 mg/kg). Body weight and blood glucose levels were assayed (Accu-Check Go, Roche) each week after the treatments. The control animals were injected with the vehicle (sterile water). Rabbits with blood glucose concentrations higher than 300 mg/dL eight weeks after the alloxan injection, and rats with blood glucose higher than 400 mg/dL three weeks after streptozotocin were considered diabetic and used for the experiments.

Animals were killed by cervical dislocation, and penectomy was done immediately. The whole penis was placed in cold Krebs buffer solution (nM composition: NaCl 118.4, KCl 4.7, CaCl_2 _2.5, KH_2_PO_4 _1.2, NaHCO_3 _25, glucose 11.7); the corpus spongiosum, tunica albuginea and the urethra were excised and discarded and longitudinal strips of corpus cavernosum were isolated and used for the *in vitro *preparation.

Animals were euthanized in accordance with the Sanofi international ethical code and international principles governing the care and treatment of laboratory animals (E.E.C. Council Directive 86/609, DJL358, 1, Dec.12, 1987) in a fully accredited AAALAC facility.

### Human tissues

Human corpus cavernosum tissues for the *in vitro *experiments were obtained from patients undergoing surgery for penile prosthesis implantation at San Gerardo Hospital, Monza, Italy. Patients had not received radiotherapy or chronic treatment with steroids, opioids or chemotherapy. Prior to surgery patients had not received PDE5 inhibitors or other vasoactive agent therapy. Specimens were available in the operating theater. Fresh tissues were collected into cold Krebs buffer solution (composition as above) and used for experiments within 24 hours. This study was approved by the ethics committee of the San Gerardo Hospital, Monza, Italy.

### *In vitro *experiments

**Figure 1:**
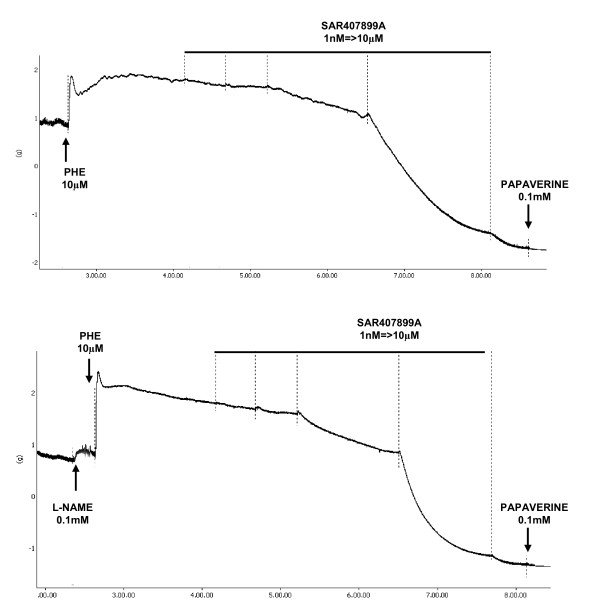
**Representative isometric recorded tracing of SAR407899 effect in isolated corpus cavernosum strips dissected from diabetic rabbits**.

Each strip was mounted in an organ bath chamber containing 20 ml of Krebs solution maintained at 37°C, constantly aerated with 95% O_2 _and 5% CO_2_, and loaded with a resting tension of 1.5 g (rat strips) or 2 g (human and rabbit strips). Changes in isometric forces were recorded using a PowerLab data acquisition system (ADInstruments, Chart 5). After a stabilization period during which the buffer solution was replaced three times, the strips were contracted to approximately 80% of the maximal contraction capacity with phenylephrine: 1 μM (rat), 10 μM (rabbit), 3 μM (human) with or without 0.1 mM L- NAME. When the response to phenylephrine was stable, cumulative concentration-relaxation response curves of SAR407899, sildenafil, Y27632 or papaverine at concentrations from 1 nM to 10 μM were constructed. At the end of the experiments papaverine (0.1 mM) was added to all preparations as reference standard for maximal tissue relaxation (100%). A representative tracing of the relaxant response of SAR 407899 in rabbit tissues is shown in Figure 1. Results were expressed as the concentration reducing the phenylephrine-induced contractions by 50% (IC_50_). The %Emax was the maximal response obtained with the compound calculated as a percentage of papaverine-induced maximal relaxation.

Emax and IC_50_, were calculated using a four-parameter logistic model according to Ratkovsky & Reedy, with adjustment by non-linear regression, using Biost@t Speed V2.0 LTS internal software. If necessary, asymptotes were constrained. The adjustment was obtained by non-linear regression using the Marquardt algorithm in SAS^® ^v9.1 software under UNIX. Values are given with their confidence intervals. For the rat isolated corpus cavernosum one-way analysis of variance (ANOVA) followed by Newman-Keuls test. For the rabbit isolated corpus cavernosum three-way ANOVA on log-transformed IC_50 _and rank-transformed %Emax was done, followed by Winer test. Analyses were carried out using SAS^® ^v8.2 for Sun Solaris via Everstat v5.0 interface. Three-way ANOVA was done Variance using SAS^® ^v9.1.

### *In vivo *experiments

Rabbits were treated either intravenously (i.v., in an ear vein) with increasing doses of SAR407899 (0.3, 1, 3, 10 mg/kg) or orally with SAR407899 (1, 3, 10, 30 mg/kg) or sildenafil (2 or 6 mg/kg). Each animal was used several times for different doses and different agents, always with a week's washout. The length (mm) of uncovered penile mucosa (penile erection parameter) was measured at different time-points, using a sliding digital caliper. The results were expressed as mean ± SEM penile length of 3-5 rabbits.

The area under the curve (AUC) was calculated for each animal in each group, and was expressed as mean ± SEM. For descriptive statistics, one-way ANOVA was done, followed by Newman-Keuls test versus the 6 mg/kg sildenafil group, using SAS^® ^v8.2 for Sun Solaris via Everstat v5.0 interface.

### Drugs

L-phenylephrine hydrochloride (Sigma Batch 7 1 K1725, stock solution 1 mM), L-NAME (Sigma Batch L0051, stock solution 100 mM), SAR407899 (batch F37096-038, stock solution 1 mM) and Y27632 (batch 066 K47001, stock solution 1 mM) were prepared in bi-distilled water. Papaverine HCl (Sigma Batch 107H1206, stock solution 10 mM) was dissolved in 2% ascorbic acid w/v in bi-distilled water. Sildenafil was either extracted from commercial 50 mg Viagra^® ^tablets, as described by Zoma [25] (the concentration of the solution was determined and analysis was done by Sanofi Discovery Analytics), or synthesized by Sanofi (SL412290, stock solution 1 mM in citric acid/DMSO/bi-distilled water 0.4/4/95.6 v/v/v). Unless otherwise specified all substances were diluted in bi-distilled water as necessary.

## Results

### *In vitro *functional activity in control-healthy and diabetic CD rats

In the *in vitro *preparation of rat corpus cavernosum, SAR407899 dose-dependently relaxed the phenylephrine pre-contracted smooth muscle to 98% of maximal relaxation, equivalent to the efficacy of papaverine (Table 1). Its potency was similar in control and diabetic animals, IC_50 _μM 0.07 and 0.05, respectively and was not significantly different from the IC_50 _of the type 5 phosphodiesterase inhibitor sildenafil, or the RhoA/Rho-kinase inhibitor Y27632. In rats with streptozotocin diabetes SAR407899 retained the same potency and efficacy as in preparations from healthy rats. Unlike SAR407899, sildenafil and Y27632 were both three to four times less potent in diabetic than controls rats, IC_50 _0.17 μM vs 0.04 μM and 0.16 μM vs 0.05 μM respectively. The maximal relaxation with sildenafil was also lower in diabetic (83%) than non-diabetic (95%) rats.

**Table 1:** In vitro relaxation of phenylephrine-pre-contracted strips from control or diabetic rats by SAR407899, Y 27632 and sildenafil

	RELAXATION OF RAT CORPUS CAVERNOSUM IC_50_, μM (95% confidence interval) %Emax ± SEM	
	
	Controls	Diabetes
**SAR407899**	**0.07**	**0.05**
	(0.03-0.17)	(0.03-0.10)
	**98 **± 2.1	**97 **± 3.1
**Y27632**	**0.05**	**0.16 **°°
	(0.02-0.09)	(0.07-0.36)
	**93 **± 1.6	**98 **± 1.5
**SILDENAFIL**	**0.04**	**0.17 **°°
	(0.001-0.23)	(0.003-0.83)
	**95 **± 4.0	**83 **± 4.3

Maximal effect is expressed as a percentage of the relaxation induced by 0.1 mM papaverine. Results were analyzed by one-way ANOVA + Newman-Keuls test. Significance of differences between groups: ○○p < 0.001 vs. CD control

### *In vitro *functional activity in SHR rats and in the same wild-type normotensive strain (WKY)

Table 2 reports the *in vitro *effects of SAR407899, sildenafil and Y27632 on phenylephrine-precontracted corpus cavernosum from SHR rats and rats from the same wild-type normotensive strain (WKY), with and without the NO-synthase inhibitor L-NAME. In WKY rats all three drugs had similar potency, IC_50 _0.10, 0.09, 0.02 μM respectively. In SHR rats, the rank order of potency of the three compounds was similar but with slightly higher IC_50 _(0.29, 0.19, 0.08 μM respectively). In presence of L-NAME sildenafil and Y 27632 were less potent both in normotensive (IC_50 _0.18, 0.61 μM vs 0.02, 0.09 μM respectively) and hypertensive rats (IC_50 _0.34, 0.56 μM vs 0.08, 0.19 μM respectively) but with a larger difference in the former (9- and 7-fold) than in the latter (4- and 3-fold). By contrast SAR407899 response was almost unaffected by L-NAME in any experimental condition.

**Table 2:** *In vitro*relaxation of phenylephrine-pre-contracted strips from normotensive WKY or genetically hypertensive (SHR) rats by SAR407899, sildenafil and Y 27632: effect of the NO synthase inhibitor L-NAME (0.3 mM)

	RELAXATION OF RAT CORPUS CAVERNOSUM IC_50_, μM (95% confidence interval) %Emax ± SEM			
	
	WKY		SHR	
	---	L-NAME	---	L-NAME
**SAR407899**	**0.10** (0.009-1.270) **89 **± 3.5	**0.13** (0.021-0.901) **86 **± 2.4	**0.29** (0.189-0.460) **94 **± 1.3	**0.28** (0.213-0.384) **94 **± 1.2
**SILDENAFIL**	**0.02**	0.18 **	**0.08**	0.34 **
	(0.014-0.060)	(0.048-0.716)	(0.039-0.155)	(0.146-0.814)
	**91 **± 3.4	**77 **± 5.7	**82 **± 3.0	**65 **± 3.6
**Y 27632**	**0.09**	**0.61**	**0.19**	**0.56**
	(0.030-0.299)	(0.373-1.025)	(0.031-1.147)	(0.272-1.185)
	**92 **± 3.2	**89 **± 3.7	**92 **± 2.5	**87 **± 2.3

Maximal effect is expressed as a percentage of the relaxation induced by 0.1 mM papaverine. Results were analyzed by one-way ANOVA + Newman-Keuls test. Significance of differences between groups: ** p < 0.003 vs. without L-NAME

### *In vitro *functional activity in control-healthy and diabetic rabbits

The superior and qualitatively different activity of SAR407899 as smooth muscle relaxant compared to sildenafil was confirmed using the *in vitro *preparation of corpus cavernosum from healthy rabbits and rabbits with alloxan-induced diabetes. This test was done with and without L-NAME (Table 3). In the absence of L-NAME, SAR407899 relaxed the corpus cavernosum of normal rabbits with similar potency and efficacy to sildenafil (IC_50 _0.40, 0.22 μM and Emax 95, 83% respectively). With L-NAME, SAR407899 had similar potency and efficacy in control and diabetic rabbits (IC_50 _0.28, 0.42 μM respectively), and its response was not affected whereas, in contrast, the potency, and particularly the efficacy, of sildenafil was drastically lower in preparations from diabetic rabbits and in the presence of L-NAME (IC_50 _1.13, 1.45 μM respectively).

**Table 3:** In vitro relaxation of phenylephrine-pre-contracted corpora cavernosa from normal (control) or diabetic rabbits by SAR407899 and sildenafil: effect of the NO synthase inhibitor L-NAME (0.3 mM)

	RELAXATION OF RABBIT CORPUS CAVERNOSUM IC_50_, μM (95% confidence interval) %Emax ± SEM			
	
	Controls		Diabetes	
	---	L-NAME	---	L-NAME
**SAR407899**	**0.40**	0.28 **	**0.42 ##**	**0.42^○○^**
	(0.23-0.69)	(0.13-0.60)	(0.20-0.90)	(0.14-1.26)
	**95 **± 1.4§	**93 **± 2.3***	**95 **± 1.4###	**96 **± 1.4^○○○^
**SILDENAFIL**	**0.22**	**1.13**	**1.45**	**1.47**
	(0.11-0.45)	(0.35-3.66)	(0.27-7.81)	(1.01-2.14)
	**83 **± 2.3	**65 **± 9.9	**65 **± 9.6	**45 ± **5.3

Maximal effect is expressed as a percentage of the relaxation induced by 0.1 mM papaverine. Results were analyzed by three-way ANOVA + Winer analysis. Significance of differences between groups: § p < 0.05 vs. sildenafil control (-L-NAME); ** 0.001 < p < 0.01 vs. sildenafil control (+L-NAME); ***p < 0.001 vs. sildenafil control (+L-NAME); ## 0.001 < p < 0.01 vs. sildenafil control (-L-NAME); ### p < 0.001 vs. sildenafil control (-L-NAME); ○○ 0.001 < p < 0.01 vs. sildenafil diabetic (+L-NAME); ○○○p < 0.001 vs. sildenafil diabetic (+L-NAME)

### *In vivo *activity on penile erection in healthy-control and diabetic rabbits

**Figure 2:**
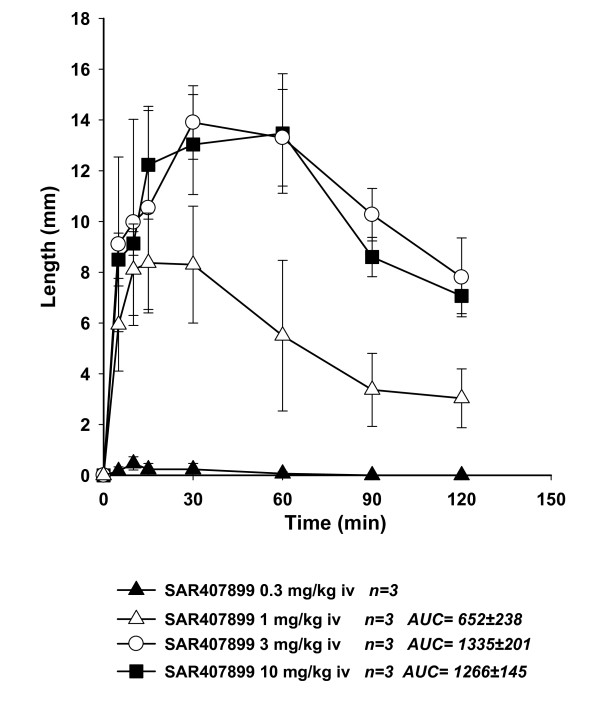
**Dose-dependent effect of intravenous SAR407899 on penile erection in normal rabbits**. Each point represents the average of three animals. Penile length in mm is plotted against the time of drug injection in minutes (AUC).

**Figure 3:**
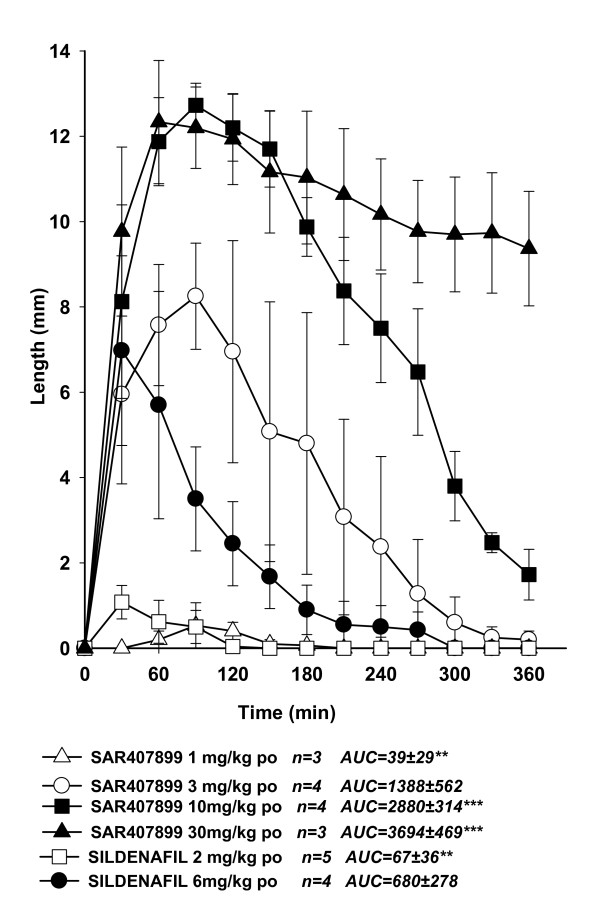
**Penile erection in normal rabbits after oral SAR407899 or sildenafil**. Each point represents the average of three-five animals Penile length in mm is plotted against the time of drug injection in minutes (AUC) 0.001 < p < 0.01 vs. sildenafil 6 mg/kg (one-way ANOVA + Newman-Keuls), *** p < 0.001 vs. sildenafil 6 mg/kg (one-way ANOVA + Newman-Keuls).

**Figure 4:**
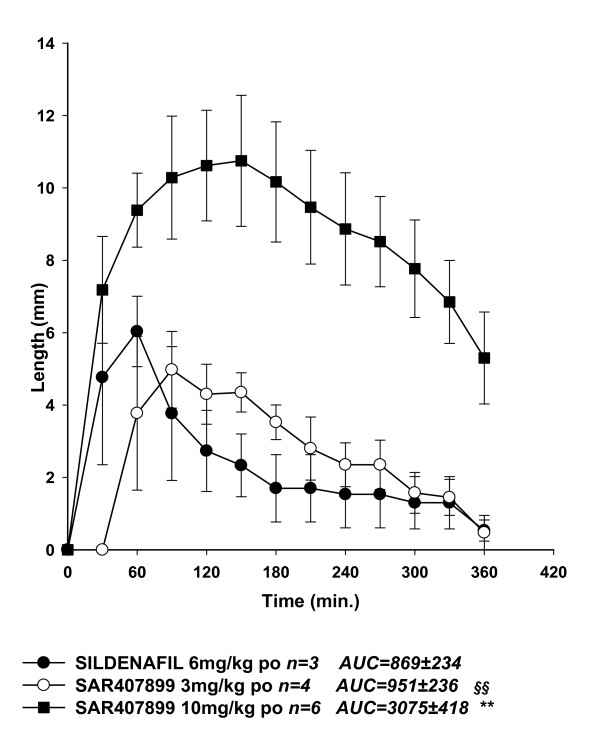
**Penile erection in diabetic rabbits after oral SAR407899 in comparison with sildenafil**. Each point represents the average of three-six animals Penile length in mm is plotted against the time of drug injection in minutes (AUC). ** 0.001 < p < 0.01 vs. sildenafil 6 mg/kg (one-way ANOVA + Newman-Keuls), §§ 0.001 < p < 0.01 vs. SAR407899A 10 mg/kg (one-way ANOVA + Newman-Keuls).

The effects of SAR407899 on penile erection *in vivo *in rabbits are shown in Figures 2, 3 and 4. Intravenous SAR407899 dose-dependently increased the length of the penis, starting from 1 mg/kg and with a maximal effect already at 3 mg/kg (Figure 2). Oral SAR407899 (3 and 10 mg/kg) also increased penile length and its effect was significantly more potent and longer lasting than sildenafil 6 mg/kg (AUC 2880 ± 314 vs. 680 ± 278). At the supra-maximal dose of 30 mg/kg, SAR407899 had still a near-maximal effect after 6 hours (Figure 3). In diabetic rabbits, oral SAR407899 (3-10 mg/kg) also dose-dependently increased penile length whereas oral Sildenafil (6 mg/kg) caused a similar increase of penile length but with less marked effects (Figure 4).

### *In vitro *functional activity in human isolated corpus cavernosum

**Figure 5:**
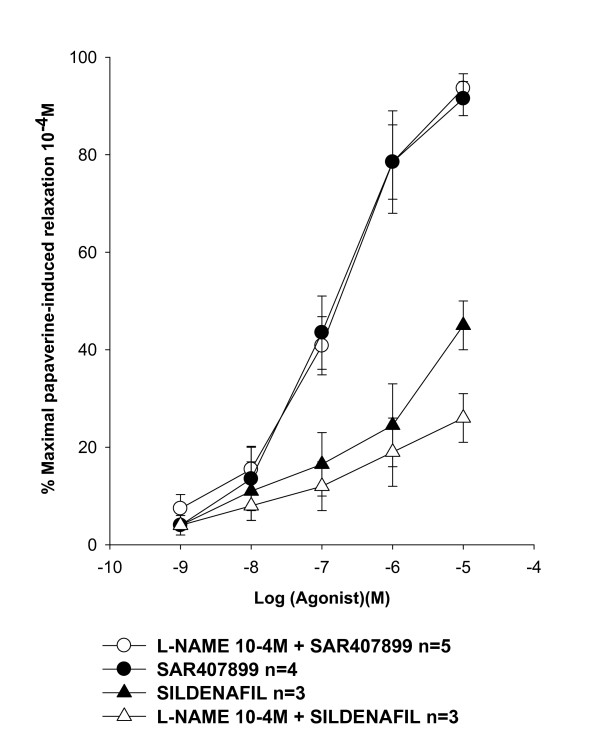
**Semilogaritmic plot of molar concentrations of SAR407899 against in vitro relaxation of human 3 μM phenylephrine-precontracted corpus cavernosum**. The relaxing effect of SAR407899A is reported on the vertical axis as a percentage of the maximal relaxation induced by papaverine (0.1 mM). The experiment was done with 0.3 mM L-NAME. Points are the mean of five different preparations.

The activity of SAR407899 was confirmed on preparations of human corpus cavernosum *in vitro *pre-contracted with 3 μM phenylephrine (Figure 5).

SAR407899 fully relaxed the corpus cavernosum smooth muscle with the same potency and efficacy with or without L-NAME (IC_50 _0.18 and 0.13 μM and max. relaxation vs. papaverine 94 and 92%). Without L-NAME sildenafil was significantly less potent and effective (IC_50 _0.51 μM, max. relaxation vs. papaverine 43%) than SAR407899. The potency, and particularly the efficacy of sildenafil was even lower in preparations with L-NAME (IC_50 _0.78 μM, max. relaxation vs. papaverine 26%).

## Discussion

SAR407899 is a highly selective Rho-kinase inhibitor that relaxes pre-contracted isolated arteries from different animal species and lowers blood pressure in rodent models of arterial hypertension [23].

In this study we examined the *in vitro *and *in vivo *actions of SAR407899 on penile tissue function in order to assess its potential value for the treatment of ED. This investigation might further contribute to understanding the importance of the Rho/Rho-kinase biochemical pathway for penile erection, particularly in diabetic patients.

SAR407899A was a potent *in vitro *relaxant of phenylephrine pre-contracted corpora cavernosa smooth muscles from rat, rabbit and man. In addition to this *in vitro *action the drug also promoted penile erection *in vivo *in rabbits with experimentally-induced diabetes, a pathology frequently associated with ED in man [2,17,22].

*In vitro*, we added phenylephrine to the bath to mimic the *in vivo *situation in which cavernosal smooth muscle contraction is maintained by α-receptor stimulation by noradrenaline released from adrenergic nerves, leading to a flaccid state of the penis. SAR407899 was fully effective and had approximately the same potency in relaxing pre-contracted cavernosal smooth muscles from control and streptozotocin diabetic or spontaneous SHR rats. The PDE_5 _inhibitor sildenafil relaxed the contracted preparations less than SAR407899 and was at least four times less potent in relaxing preparations from diabetic and SHR rats, compared to normal rats. The difference between the potency of SAR407899 and sildenafil in relaxing preparations from diabetic and healthy animals was confirmed, and even magnified, in rabbit corpora cavernosa. In these experiments, SAR407899 showed the same potency and efficacy in preparations from healthy and alloxan diabetic rabbits while sildenafil was much less potent and effective in diabetic rabbits.

It is important to recall that Rho-kinase is highly expressed in cavernosal smooth muscle cells of man and other mammals [14] and is up-regulated in corpora cavernosa of aging and diabetic animals, and animals with spontaneous hypertension [17-19]. The hyperfunction of this signaling pathway, which suppresses endothelial nitric oxide synthase (e-NOS), may be one mechanism leading to ED associated with aging, diabetes and cardiovascular hypertension [17,18,22,26]. Consequently, inhibition of this pathway by a selective Rho-kinase inhibitor like Y-27632 improved ED in aging and diabetic rodents [16,20,21].

In our study Y-27632, used as reference compound for the activity of SAR407899 on rat corpus cavernosum, relaxed this preparation with potency and efficacy similar to that of SAR407899. Unlike SAR407899, however, it was slightly less potent in relaxing corpora cavernosa from streptozotocin diabetic rats than from normal rats. It is hard to explain this difference between two compounds believed to act with a common mechanism of action. Since basal release of NO from the endothelium seems to be present in organ chamber setting as reflected by sildenafil and L-NAME effects, the impact of NOS inhibition on Y27632 could be explained by the loss of its effects through Rho-kinase inhibition-mediated NOS activation. Alternatively the superior selectivity of SAR407899 over Y-27632 as Rho-kinase inhibitor might at least partially explain it [23].

It has been reported that Rho-kinase antagonism stimulates penile erection in rats by a mechanism that is not primarily dependent on the NO pathway which, instead, is required for the activity of PDE_5 _inhibitors [11,19]. Therefore, to confirm the specificity of SAR407899 as Rho-kinase antagonist in the animal models of ED, we tested its ability to relax corpora cavernosa of normotensive and hypertensive rats and of normal and diabetic rabbits in the presence of the NO-synthetase inhibitor, L-NAME. As expected, SAR407899 was equally effective as a relaxing agent with and without L-NAME in all experimental conditions, while sildenafil was from four to eight times less active in the presence of L-NAME in preparations from normotensive and hypertensive rats, and in those from healthy and diabetic rabbits. It is noteworthy that sildenafil had similarly lower potency in diabetic than healthy rabbit preparations with L-NAME. This suggests that sildenafil is largely dependent on NOS activity in corpus cavernosum relaxation. We confirmed that SAR407899, unlike sildenafil, also acts through the same mechanism on human tissue, where its potency and efficacy *in vitro *on phenylephrine-precontracted corpora cavernosa with and without L-NAME were similar.

All these results point to different molecular mechanisms for ED in healthy and diabetic animals and suggest that SAR407899, by selectively acting on the RhoA/Rho-kinase pathway, might be more effective than sildenafil and other PDE_5 _inhibitors in improving ED in diabetic patients. This conclusion is further supported by the *in vivo *results with SAR407899 in normal and alloxan-induced diabetic rabbits. The superior potency of SAR407899 over sildenafil was evident when the compound was given orally to non-diabetic rabbits. Peak effects in inducing penile erection were similar after 3 mg/kg SAR407899 and 6 mg/kg sildenafil, but the effect of the former began later and lasted much longer, confirming its oral bioavailability and long-lasting action already reported in other species [23]. Most important, SAR407899, unlike sildenafil, retained at least the same potency and efficacy in diabetic rabbits. This was clear from the similar or even larger AUC in the plot of penile length against time at the oral dose of 10 mg/kg SAR407899.

## Conclusion

This study showed that the highly selective Rho-kinase inhibitor SAR407899 is a relative potent relaxing agent of corpora cavernosa from different animal species and man. These results, in stimulation of penile erection, may be useful in the prevention and therapy of a number of erectile dysfunctions, particularly those depending on hyper-functioning of the RhoA/Rho-kinase system, such as diabetes and hypertension. Future studies are required to confirm the potential of this compound and other more powerful molecules for ED.

## Competing interests

F.Guagnini, M.Ferazzini, T.Croci are employees of sanofi R&D a pharmaceutical company engaged in discovery, development and distribution of therapeutic solutions. M.Grasso and S.Blanco are employees of San Gerardo Hospital that supplies human tissues. All the experiments were founded and performed in sanofi laboratories.

## Authors' contributions

FG, MF and TC designed the experiments, performed animal and human experiments and drafted the manuscript. MG, SB supplied human tissues and participated in drafting the manuscript. All authors have read and approved the final manuscript.
